# Youtube as an Information Source During the Coronavirus Disease (COVID-19) Pandemic: Evaluation of the Turkish and English Content

**DOI:** 10.7759/cureus.10795

**Published:** 2020-10-05

**Authors:** Ömer Ataç, Yunus Can Özalp, Rifat Kurnaz, Osman Murat Güler, Melikşah İnamlık, Osman Hayran

**Affiliations:** 1 Public Health, School of Medicine, Istanbul Medipol University, Istanbul, TUR

**Keywords:** covid-19, discern, mici, video power index, youtube

## Abstract

Introduction: YouTube is an important online source of information and has two billion users globally. Its viewing numbers tend to increase exponentially in extraordinary global situations. Our aim in this study was to review and evaluate the contents of the most frequently viewed YouTube videos during the Coronavirus disease 2019 (COVID-19) pandemic.

Methods: In this qualitative study, contents of the most frequently viewed Turkish and English YouTube videos regarding the COVID-19 pandemic were examined and scored with modified DISCERN, medical information and content index (MICI), and video power index (VPI) during April 2020.

Results: The mean DISCERN score of Turkish videos was similar to that of English videos (2.55±1.40 and 2.43±1.25, respectively). The total MICI score tended to be higher in Turkish videos. News channels released 86.9% of all 168 videos and 65.2% of all 23 misleading videos. When the descriptive characteristics of videos were compared in terms of their content category, average view counts, view ratios, and VPIs of misleading videos were higher than those of the useful videos. Only, the likes ratio of useful videos was higher than that of the misleading videos.

Conclusions: Since there is no peer-review system on YouTube, people can almost release every type of video. It is very important for the content of videos that are released through news channels to be accurate because the important messages can be spread among people in society through them. In our study, especially some Turkish videos included many different rumors and faulty statements. During the extraordinary situations such as the pandemic, the videos of official health authorities and international institutions should be more visible on YouTube.

## Introduction

Coronavirus disease 2019 (COVID-19) was primarily announced to the world with cases of unknown etiology pneumonia from the city of Wuhan located in the state of Hubei, China [1]. The situation report of the World Health Organization (WHO), which was published on January 30, 2020, described the interim name of the disease "2019-nCoV acute respiratory disease" and "2019-nCoV" as an interim name of the virus [2]. On February 5, 2020, the name of the virus was announced as Severe acute respiratory syndrome coronavirus 2 (SARS-CoV-2) by The International Committee on Taxonomy of Viruses (ICTV), and the disease that the virus leads to was called COVID-19 in a press conference of the WHO Director-General [3-5]. While the outbreak of the disease was initially identified as Public Health Emergency of International Concern on January 30, it was later announced as a pandemic on March 11 [6,7]. By the first week of April, the confirmed cases exceeded one million and reached 212 different territories [8]. As of April 18, the number of confirmed cases and death were 2,160,207 and 146,088, respectively [9]. The first confirmed case in Turkey was seen on March 10, and the first death took place on March 15. As of April 18, the number of confirmed cases and deaths is 82,329 and 1890, respectively [10].

The virus spreads from person to person at close range through droplets. The other common type of contagion is the spread through mucosa with contaminated hands [11]. The transmission ability of asymptomatic cases is one of the difficulties in a pandemic. Since a vaccine has not been developed yet, personal protective measures such as hand-washing, physical and social distancing, and isolation of the infected individuals are the most effective ways to struggle against pandemic [11]. People need to access correct information and then apply these protective measures in their lives to achieve this. Study results indicate that social media and online platforms on the internet are the major sources of medical information for many people [12,13]. It is important to access correct and reliable information through these channels. Unfortunately, almost none of these platforms or social media are peer-reviewed, and they may include a lot of false or misleading information. 

YouTube is an important online source of information with its two billion users globally. Its viewing numbers tend to increase exponentially in extraordinary global situations [14]. Not only ordinary people or patients but healthcare institutions and professionals also use and share information via YouTube [15]. Because of the open access and lack of peer review, there are some concerns with regard to reliability, confidentiality, and privacy of contents [16,17]. It is prone to misinformation, disinformation, and anecdotes which are not based on any evidence [13,18]. In the literature regarding past pandemics, it was demonstrated that the percentage of videos that contain false information is between 8.0% and 23.8% on YouTube [19-21]. 

The content analysis regarding social media and online platforms has been a significant research issue in recent years. The spreading of health information through the internet is crucial, especially during extraordinary times such as disease pandemics [20,22,23]. Our aim in this study was to review and evaluate the contents of the most frequently viewed YouTube videos during the COVID-19 pandemic.

## Materials and methods

This study was conducted as a qualitative study. Contents of the most frequently viewed Turkish and English YouTube videos regarding the COVID-19 pandemic were examined throughout April 2020.

Selection of the study material

**Figure 1:**
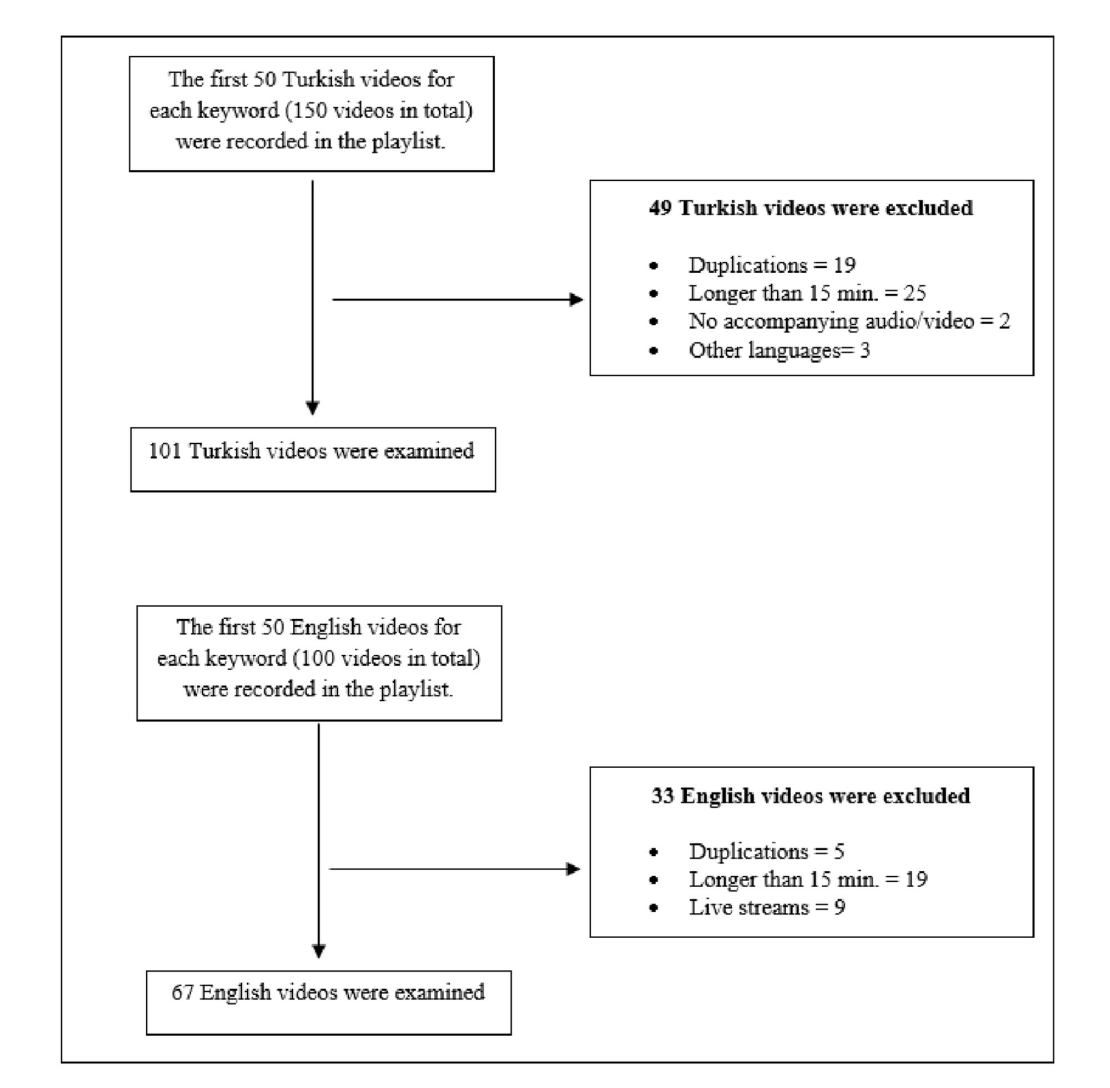
Flow diagram for the selection process

On April 9, 2020, the search process was conducted on YouTube by using both Turkish and English keywords such as ‘’Corona virüsü’’, ‘’Koronavirüs’’, and ‘’Koronavirüs Hastalığı’’; and ‘’COVID-19’’ and ‘’Corona virus’’. In order to prevent the influence of cache, cookies, and watch history on the search process, a new YouTube account was created for this study. In all searches, the relevancy level in filter was selected as default on YouTube. The first 50 results were recorded in a separate list based on each keyword. The reason why the first 50 results were selected is that some studies show that YouTube users do not tend to watch videos after a couple of pages [24]. These videos are reviewed and examined in accordance with the stages in Figure 1. Since the literature demonstrates that the optimal length for a YouTube video is between 10 and 16 minutes, those that exceeded the 15-minute threshold were eliminated during the study [25]. Ultimately, 101 Turkish and 67 English videos, which met these criteria, were included in the study. Since this study was conducted through open data that are accessible to all people, any ethics committee approval was not taken.

Evaluation of the contents

The descriptive characteristics, such as the name of videos, their upload dates, view counts, likes, sources, and content, were recorded on April 10, 2020. Every video was evaluated through the principles of DISCERN and medical information and content index (MICI). Moreover, a video power index (VPI) was calculated for each video, and the evaluations of modified DISCERN and MICI were conducted by researchers [20,22,26,27].

Modified DISCERN is a five-question scale that was adapted by Singh et al. from a 16-question DISCERN tool, which was developed by Charnock et al. [27,28]. Each criterion is ranked as 1-0 (yes/no) and scored between zero and five (Table 1).

**Table 1:** Modified DISCERN

1. Are the aims clear and achieved?
2. Are reliable sources of information used? (i.e., publication cited, speaker is a certified physician)
3. Is the information presented balanced and unbiased?
4. Are additional sources of information listed for patient reference?
5. Are areas of uncertainty mentioned?

MICI was developed by Nagpal et al. during the period of the Ebola epidemic in order to evaluate the content quality of videos that contain medical information and has been used by studies about the COVID-19 [20,22]. It examines every video under these main categories: prevalence, transmission, signs and symptoms, screening/testing, and treatment/outcome. Each main category includes five different criteria, which means that there are 25 different criteria in MICI. Every criterion is ranked as 1-0 and scored between zero and 25 (see Appendices).

VPI ([(view ratio x like ratio/100]), where view ratio=views/day and like ratio=[(likes x 100)/ (likes + dislikes)] was developed as an index by Erdem and Karaca in order to measure the power of social media based on the descriptive features of videos and has been used by different studies [12,26].

While two researchers (Mİ and YCÖ) evaluated the Turkish videos, two other researchers (RK and OMG) examined the English videos separately for eligibility. A third researcher (ÖA) was consulted during the evaluation process when there was a conflicting issue to finalize the decision. The level of agreement between researchers was significantly high for both languages (Cohen’s kappa: 0.81 for Turkish, and 0.85 for English).

The content evaluation was conducted under three categories: useful, misleading, and news update [20,21,29]. Those videos which contain scientific and reliable information were coded as useful. In contrast, the ones which include false information, conspiracy theory, or manipulation were coded as misleading. Finally, those that share information through new channels were coded as news update. Each video belonged to only one category.

Statistical analysis

After the data were coded in Microsoft Office 365 Excel (Microsoft, Washington, USA), they were transferred to SPSS 24.0 (IBM, New York, USA) for analyses. After the normality examinations, mean, standard deviation, frequencies, and percentages were calculated for descriptive statistics, unpaired T-test was used to analyze the differences. The statistically significant level was accepted as p< 0.05.

## Results

The total view count of all 168 videos is 67,222,756. As shown in Table 2, the time interval between the release date of English videos and the date of this research is shorter than the case of Turkish videos (p< 0.001). 

**Table 2:** Descriptive characteristics of Turkish and English videos

Descriptive characteristics	Turkish (n=101) mean±SD	English (n=67) mean±SD	p
Days since release date	28.38±22.78	4.22±7.66	<0.001
Number of views	373,496.83±673,947.92	511,508.78±635,301.38	0.079
Views/day	15,606.50±25,034.16	194,348.33±307,960.44	<0.001
Number of likes	12,764.68±33,054.33	6923,89±21.825,83	0.183
Number of dislikes	463.42±2061.24	816.89±1235.28	0.214
Number of comments	689.38±2284.22	2641.29±3200.62	<0.001
Length of duration (sec)	227.87±180.94	359.94±212.35	<0.001
VPI score	93.24±198.09	1603.19±2304.32	<0.001
Modified DISCERN score	2.55±1.40	2.43±1.25	0.556

The views/day ratio of English videos was significantly higher than that of Turkish ones (p< 0.001). The number of comments, length of duration, and VPI scores of the English videos were significantly higher than those of the Turkish videos, whereas the mean DISCERN score of Turkish videos was similar to that of the English videos (2.55±1.40 and 2.43±1.25 respectively). Mean MICI scores for Turkish and English videos are presented in Table 3.

**Table 3:** MICI scores of the videos by language

	Turkish mean±SD	English mean±SD	p
Prevalence	0.66±1.44	1.07±1.31	0.062
Transmission	1.03±1.40	0.61±1.18	0.046
Signs-Symptoms	1.11±1.64	0.24±0.78	<0.001
Screening/Testing	0.11± 0.31	0.16±0.73	0.501
Treatment/Outcome	0.42±0.87	0.67±1.11	0.114
Total MICI Score	3.33±3.09	2.76±2.49	0.212

Total MICI scores tended to be higher in Turkish videos; however, there was no significant difference between the two languages (p=0.212). Mean scores for transmission and signs-symptoms of Turkish videos were significantly higher than those of the English videos (p=0.046 and p< 0.001, respectively). Of all 168 videos, 86.9% were released in news channels (Table 4).

**Table 4:** Distribution of videos by language, source of release, and content

Source of release	Turkish (n=101)			English (n=67)		
	Useful (%)	Misleading (%)	News Update (%)	Useful (%)	Misleading (%)	News Update (%)
Ministry/academic/hospital (n=4)	4 (8.7)	0	0	0	0	0
News channels (n=146)	35 (76.1)	9 (56.3)	39 (100.0)	14 (82.4)	6 (85.7)	43 (100.0)
Other (n=18)	7 (15.2)	7 (43.7)	0	3 (17.6)	1 (14.3)	0
Total	46 (100.0)	16 (100.0)	39 (100.0)	17 (100.0)	7 (100.0)	43 (100.0)

About 33.6% of news channels videos were categorized as useful. Around 15.8% of Turkish videos and 10.4% of English videos had misleading content, respectively. Of all 23 misleading videos, 65.2% were released by news channels. When the descriptive characteristics of videos were compared in terms of their content category, it was found out that the average view counts, view ratios, and VPIs of misleading videos were higher than those of the useful videos, but the difference between these groups was not statistically significant (Table 5). Only the likes ratio of useful videos was higher than that of the misleading videos.

**Table 5:** Content evaluation of videos by descriptive characteristics

Descriptive characteristics	Video categories		
	Useful (n=63)	Misleading (n=23)	p
Mean number of views	404,043.71±726,972.38	642,000.57±943,693.07	0.219
Mean length of duration (sec)	287.83±223.09	344.70±229.96	0.302
Likes ratio	89.10±15.69	80.22±27.35	0.204
View/day	40,694.24±80,177.21	99,054.95±189,597.07	0.165
VPI	356.88±685.93	859.30±1731.62	0.188

## Discussion

Our study findings indicated that only 37.5% of the reviewed videos have useful content. A recent study that examined videos in English and Chinese regarding the COVID-19 pandemic found the proportion of useful content as 58.8% [20]. In another study about H1N1 influenza, the proportion of useful video content was 61.3% [21]. Both studies have indicated higher proportions of useful content compared to our study. The reason for the low proportion of useful content could be the high proportion of misleading content in Turkish videos than in English videos (15.8% and 10.4%, respectively) in our study group. On the other hand, 65.2% of misleading videos were released by news channels.

In both languages, the majority of the videos (86.3%) were released by the news agencies. There was no video from institutions such as WHO, Centers for Disease Control and Prevention (CDC), or European Centre for Disease Control and Prevention (ECDC) in our study. While Khatri et al.'s study included only six (5.3%) videos from the WHO, another study conducted by Pathak et al. did not contain any video from the WHO or CDC [20,29]. Four Turkish videos, one by the Turkish Ministry of Health, one by an academic institution, and two by hospitals, were released by professional health institutions. One reason for this finding can be the professional health institutions' lack of interest in electronic media. It may also depend on YouTube's algorithm of favoring some mainstream media channels.

The difference between the mean DISCERN scores of Turkish and English videos was not statistically significant. The mean scores found by Khatri and his colleagues are higher than our findings [20]. In the results of MICI, the average score of Turkish videos was higher than that of English videos. In Khatri and his colleagues' study, the average scores of both English and Chinese videos are higher than our study results [20]. The difference in the ranks of English videos throughout the time from Khatri et al.'s study to ours may have led to differences in the scores of DISCERN and MICI when two studies compared [20]. On the other hand, there might be a bias of researchers in terms of their evaluations and rankings. Nevertheless, it is still significant to see that only six videos had a score of 10 or above in a ranking system that examines the information quality of content, in which the maximum score is 25. Furthermore, four of those videos were released through news channels, and none of them was produced by an academic institution or the Ministry of Health. During the COVID-19 period, one research studied the most popular 100 videos in English to examine the information quality of preventive behaviors and indicated that only one-third of those videos included one of the seven different preventive behaviors. Moreover, it ascertained that 79.0% of all videos had content that could trigger fear and anxiety in society [23].

In our study, especially some Turkish videos included many different rumors and faulty statements such as; "the virus is developed in a laboratory environment, its treatment is certain, but they're waiting for the right time to announce it". We noticed that even some medical doctors expressed misleading or faulty comments such as "number of cases will not increase", "it was a virus that should not be feared", "saltwater gargle, vinegar water, or kelle paca soup (a traditional dish consists of a sheep's head and trotters) prevents this disease", or even "the virus did not exist at all". In Turkey, the YouTube videos regarding the COVID-19 are provided through COVID-19 health portals, and these portals are linked to the information address of the Ministry of Health [10].

Limitations

There are some limitations in our study. This study was conducted in a specific time period; it can lead to different results if it is conducted in different periods because the content and definitive features of YouTube are subject to change constantly. However, as this study was conducted for more than three months after the announcement of the first confirmed case, the videos which were analyzed might relatively have a standard ranking. Although the kappa coefficient was used for the measurement of DISCERN and MICI scores, there might be some issues regarding both intra- and inter-observer bias in our study. Lastly, it might also be a limitation that we only included videos in two different languages with five different keywords, which led us to evaluate 50 videos for each keyword.

## Conclusions

YouTube is one of the most common news and information source in today’s world because of its simple access and provision of various contents. However, YouTube does not provide a peer-review agent, except for copyright and common complaint issues, people can almost release any types of videos. Consequently, it also becomes a suitable platform for the spread of misinformation and disinformation. News channels are the most-viewed sources of videos for users. It is very important for the content of videos that are released through these news channels to be accurate so that the important messages can be spread among people in society through them. However, the fact that the videos created by international institutions, academic and ministry accounts tend to be watched less than news channels shows that these institutions are not successful in using such platforms. Different solutions should be developed in order to increase the view counts of these institutions. During the extraordinary situations such as pandemics, the videos of official health authorities and international institutions should be more visible on YouTube.

## Appendices

**Table 6:** Set of criteria used for scoring within each MICI component

Prevalence	Each item is given one point if mentioned in the video. Maximum score is five. Number of confirmed cases reported, number of suspected cases reported, number of deaths reported, number of countries involved, number/proportion of patients who are severely ill
Transmission	Each item is given one point if mentioned in the video. Maximum score is five. Location of origin of virus, zoonotic transmission (i.e., contact with animals), human to human transmission, incubation period, transmission route via droplets (include: precautionary measures of wearing mask, handwashing)
Signs and Symptoms	Each item is given one point if mentioned in the video. Maximum score is five. Fever, upper respiratory tract symptoms (cough, sore throat, runny nose), lower respiratory tract symptoms (pneumonia)/shortness of breath), myalgia-arthralgia-lethargy, diarrhea
Screening Testing	Each item is given one point if mentioned in the video. Maximum score is five. Mentions there is a test available, mentions the test uses respiratory secretion to test, mentions that PCR can be used for identification, shows how this test is done, mentions criteria for testing/screening
Treatment/Outcome	Each item is given one point if mentioned in the video. Maximum score is five. Mild symptoms can be self-resolving, some patients become ill (mentions hospitalization, ICU admission), can be dangerous or lead to death, treatment is supportive, vaccination not currently available
